# In Silico Methods for Identification of Potential Active Sites of Therapeutic Targets

**DOI:** 10.3390/molecules27207103

**Published:** 2022-10-20

**Authors:** Jianbo Liao, Qinyu Wang, Fengxu Wu, Zunnan Huang

**Affiliations:** 1 Key Laboratory of Big Data Mining and Precision Drug Design of Guangdong Medical University, Key Laboratory of Computer-Aided Drug Design of Dongguan City, Key Laboratory for Research and Development of Natural Drugs of Guangdong Province, School of Pharmacy, Guangdong Medical University, Dongguan 523808, China; 2 The Second School of Clinical Medicine, Guangdong Medical University, Dongguan 523808, China; 3 Hubei Key Laboratory of Wudang Local Chinese Medicine Research, School of Pharmaceutical Sciences, Hubei University of Medicine, Shiyan 442000, China; 4 Marine Biomedical Research Institute of Guangdong Zhanjiang, Zhanjiang 524023, China

**Keywords:** drug discovery, target identification, binding site, druggability, therapeutic target

## Abstract

Target identification is an important step in drug discovery, and computer-aided drug target identification methods are attracting more attention compared with traditional drug target identification methods, which are time-consuming and costly. Computer-aided drug target identification methods can greatly reduce the searching scope of experimental targets and associated costs by identifying the diseases-related targets and their binding sites and evaluating the druggability of the predicted active sites for clinical trials. In this review, we introduce the principles of computer-based active site identification methods, including the identification of binding sites and assessment of druggability. We provide some guidelines for selecting methods for the identification of binding sites and assessment of druggability. In addition, we list the databases and tools commonly used with these methods, present examples of individual and combined applications, and compare the methods and tools. Finally, we discuss the challenges and limitations of binding site identification and druggability assessment at the current stage and provide some recommendations and future perspectives.

## 1. Introduction

**Figure 1:**
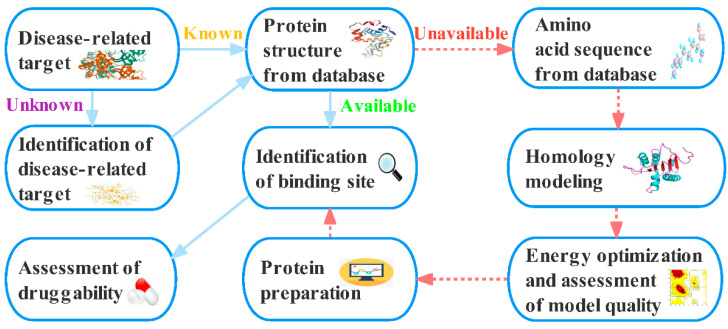
Flow chart of computer-based identification of potential drug targets.

In medicinal chemistry, researchers are interested in identifying vital proteins whose biological functions are clearly linked to disease, and these proteins become targets for medication development. Drug development must pass through specific stages consisting of disease-related information collection, target identification, target validation, lead discovery, lead optimization, preclinical studies, and clinical trials [1,2,3]. The traditional target identification process identifies binding sites and molecular binding modes through experimental methods, which is time consuming and expensive [4,5]. With the development of bioinformatics and proteomics, computer-aided drug target identification methods have attracted increased attention. By converting target information into computer language and performing data computation and analysis, computer-aided drug target identification methods not only greatly reduce the searching scope of experimental targets but also shorten the research cycle and reduce the experiment cost. With the acquisition of information over time, prediction accuracy may be improved and more closely reflect experimental results. The current process of computer-based potential drug target identification is shown in Figure 1.

Computer-based target identification mainly involves disease-related target identification, binding site identification, and druggability evaluation.

In this review, we will focus on the identification of binding sites and assessment of druggability during the target identification process (for the identification of disease-related targets, please refer to the review written by Zhang X et al. [3] in our research group). The binding site can be used to determine whether the target protein and the ligand can interact [5]. The identification of binding sites can exclude target proteins or binding sites that have weak or no binding ability to ligands. In addition, identifying binding sites is not only beneficial for the functional characterization of proteins but also provides the knowledge about them to guide the design of inhibitors and antagonists [6]. On the other hand, the assessment of druggability is performed to determine whether the target protein has the ability to interact with drug-like molecules and exert a therapeutic effect [7]. This stage is important considering that up to 60% of failures in the clinical trial phase may be attributed to non-druggable targets [8,9]. Early druggability assessment can address the issue of low utility or druggability for most proteins in the clinical trial phase. Furthermore, it is a prerequisite for screening whether a target protein can be used for treatment. The identification of binding sites and assessment of druggability are essential for protein function annotation, cellular activity mechanism elucidation, molecular docking, and rational drug design.

**Figure 2:**
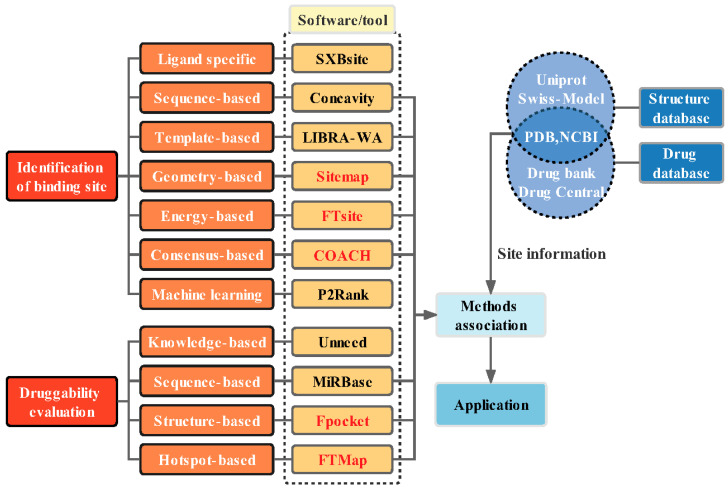
Overview of binding site identification and druggability evaluation.

In this review, we summarized the methods of binding site identification and druggability evaluation, including rationale, known characteristics, advantages, and disadvantages. In addition, various databases (resource databases, probe databases, and benchmark datasets for method testing) and some frequently used software and tools were classified. In order to have a better understanding of the presented methods, some examples showing the applications of these tools were provided. The predictive accuracy and advantages were established by comparing these tools, and their potential in computer-based target identification was analyzed. The overall outline of this review is illustrated in Figure 2.

Software or tools in red are introduced in this article.

## 2. Methods for Target Identification

Most of the current drug targets are proteins [10], and the 3D structures of these proteins need to be known in target identification. Some 3D protein structures can be extracted from databases. For proteins whose 3D structures cannot be obtained directly from databases, homology modeling can be performed to obtain their 3D structures by extracting their amino acid sequences from databases and identifying protein sequences with high homology through similarity searching and sequence matching. The accuracy of predicting binding sites and druggability based on structures through homology modeling has been demonstrated to be satisfactory [11,12,13,14]. The available sites can initially be selected using binding site identification methods, and druggable sites with a high confidence degree can be selected from the available sites using druggability evaluation software. The presented target identification methods in this review include the strategies and rationales for binding site identification and druggability assessment, which are described in several sections.

### 2.1. Binding Site Identification

The biochemical functions of proteins are determined by the mode and type of interactions with other molecules. Usually, not all residues are involved in protein interactions, and the location where the interaction takes place is defined as the binding site. The identification of the binding site is performed by analyzing the physicochemical and shape characteristics of the protein region, which is a key step in the discovery of new chemical entities in molecular docking for structure-based drug design [15].

**Figure 3:**
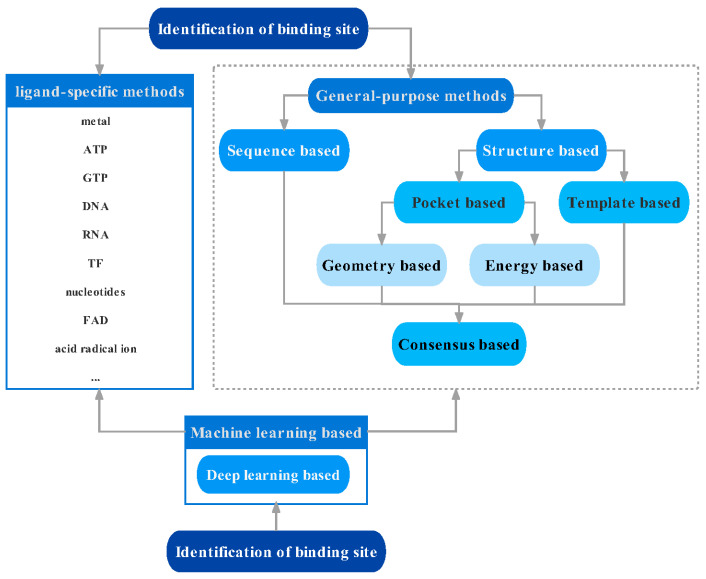
Classification of methods for the identification of binding sites.

The classification of binding site identification methods is shown in Figure 3. The methods of binding site identification include ligand-specific methods and general-purpose methods [16]. General-purpose methods can be divided into two categories: sequence-based and structure-based methods, which can be further divided into template-based and pocket-based methods. Furthermore, pocket-based methods can be divided into geometry-based and energy-based methods. The consensus-based methods are a prediction algorithm procedure that can combine any of the geometry-based, sequence-based, energy-based, and template-based methods. Machine learning-based methods including deep learning-based methods can be used to complement ligand-specific and general-purpose methods.

#### 2.1.1. Ligand-Specific Methods

**Figure 4:**
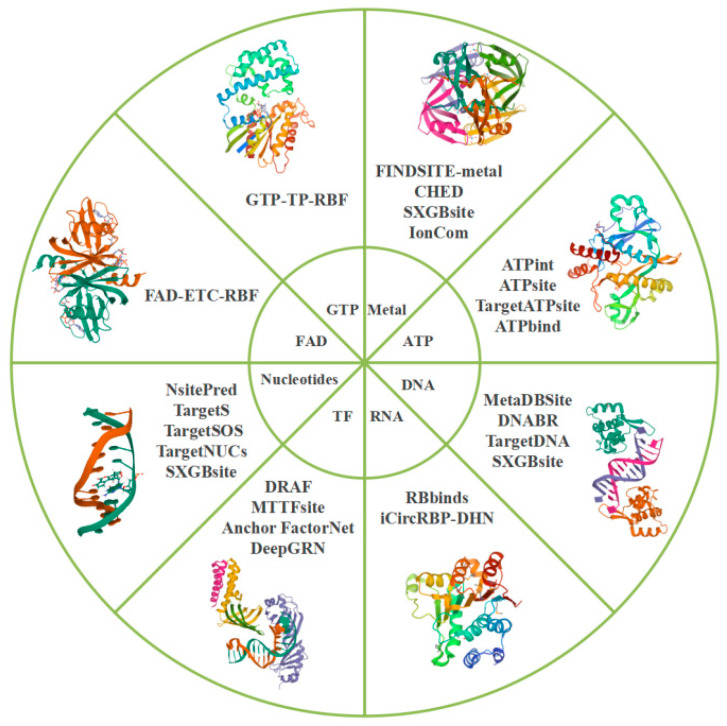
Tools used in ligand-specific methods to predict binding sites.

Ligand-specific methods are a group of methods typically used for ligand-type prediction. Due to the specific role, size, and distribution of protein–ligand interactions, various types of ligands tend to bind to different categories of residues with high specificity, and the accuracy of predicting the binding sites of the corresponding type of the ligands is usually high, while the accuracy of predicting the binding sites of other types of ligands is low. [16]. There are various ligand-specific predictors for predicting the binding sites (or pockets) of various ligand types. For instance, CHED [17], FINDSITE-metal [18], and IonCom [19] are used in the prediction of metal ion-binding sites based on the principle that four specific amino acids (Asp, Cys, Glu, and His) preferentially bind to transition metal ions. Consequently, sites that satisfy the binding distance criteria can be found by searching the triad of these amino acids at all positions of the 3D structure [17,18,19]. Tools for DNA-binding sites, such as MetaDBSite [20], DNABR [21], TargetDNA [22], and DRNApred [23], rely on features such as sequence conservativeness, predicted secondary structure, predicted solvent accessibility, electrostatic potential, and hydrophobicity, which have been shown to play an important role in predicting protein–DNA interactions. Differences between DNA-binding and non-DNA-binding residues can be quantified by assigning different weights. Ultimately, DNA-binding sites and non-DNA-binding sites can be identified based on the score [20,21,22,23]. On the other hand, tools for predicting nucleotide-binding sites, including TargetS [24], TargetSOS [25], and TargetNUCs [26], are used to extract characteristic vectors, such as the position-specific scoring matrix, and determine protein secondary structures, which have been shown to be important in the prediction of nucleotide-binding residues. These distinctive features, i.e., predictors, can differentiate nucleotide-binding residues and non-nucleotide-binding residues. Different predictors can be assigned different weighted scores to distinguish nucleic acid-binding sites and non-nucleic acid-binding sites [24,25,26]. As different ligands have the tendency to specifically bind to different types of residues, ligand-specific algorithms are more suitable than general-purpose methods for predicting binding sites according to the ligand type [16]. The commonly used tools in ligand-specific methods for predicting ligand-binding sites are shown in Figure 4. However, in this review, we would not focus on tools based on ligand specificity as they have a narrower scope of application in target identification.

#### 2.1.2. General-Purpose Methods

In comparison with ligand-specific methods, general-purpose methods are more suitable for the prediction of broad binding sites. Their predictive power is greater and not restricted to ligand prediction. Therefore, they are more commonly chosen for the identification of binding sites [16]. This section describes the available general-purpose methods.

##### Sequence-Based

Sequence-based methods are mostly based on the principle of residue conservation, such as ConCavity [6] and MPLs-Pred [27]. Molecule-binding residues are assumed to be functionally significant, and binding sites are analyzed by residue conservation analysis, for which conserved amino acids in multiple sequence alignment columns of homologs are the most common source [6,26]. The advantage of this approach for prediction is the identification of binding sites composed of relatively conserved residues. However, the disadvantage is a higher number of false positives because amino acid sequence conservation is not a sufficient condition for evaluating binding sites, and many non-binding residues are also often conserved [6]. Moreover, sequence conservativeness is known to correlate closely with ligand-binding and catalytic sites but less so with residues in the protein–protein interface [28]. In contrast to structure-based methods, sequence-based methods do not take into account the geometric structure and physicochemical properties of binding sites, and thus their accuracy of predictions is usually low [29].

##### Structure-Based

Structure-based methods are divided into two types: template-based and pocket-based [15]. Template-based methods predicts the binding site of a target protein structure by obtaining the binding site characteristics of the template protein structure, which would have high amino acid sequence similarity to the target protein. Pocket-based methods rely on the geometric and energetic properties of the modeled structures.

In the template-based approach, binding sites of target proteins are obtained from template proteins with high homology found through a sequence matching procedure in a database when the structures of the target and template proteins are both known, such as LIBRA-WA [30] and bSiteFinder [31]. It is based on the principle that structurally similar proteins have similar functions; thus, the structures of the binding sites are similar [30,31,32]. Given the structural similarity of the template and target proteins, it is easy to predict the possible binding sites on the target protein structure using the characteristics of known binding sites as a reference. The advantage of this approach is that it can be used even when the structure of the target protein is not available in a database as long as the amino acid sequence is known. Template-based methods are more effective for targets with highly conserved binding sites, whose prediction success rate can reach up to 70%; however, the rate for non-conserved targets is as low as 33% [33]. Moreover, if the binding sites of template proteins have been experimentally confirmed to exist, template-based methods would be appropriate for predicting the binding sites of target proteins with high accuracy according to the binding sites of template proteins. Despite the high prediction rate, the performance of template-based methods is dependent on the global or local structural similarity, which is further caused by sequence similarity of the binding sites of template proteins [34]. Therefore, the predicted binding sites are more likely to be binding sites of template proteins, and these methods cannot be used to predict the binding sites of novel targets, thus making them inferior to pocket-based methods.

Pocket-based methods can be classified as geometry-based and energy-based methods. Geometry-based methods can be divided into α-shape based, sphere-based, and grid-based methods by identifying the different surface states of the protein. For example, Fpocket [35] and CASTp [36] explore the cavity and void size of the surface based on the α-shape form. In addition, the size, burial, and flexibility of binding sites are known key factors in binding site identification [5]. The largest and deepest cavity is identified as the ligand-binding site by measuring its size. A geometry-based tool, Sitemap [7,37], focuses on hydrophobicity and hydrophilicity as high hydrophobicity and low hydrophilicity tend to provide solvent protection and a better ligand-binding space [7]. The accuracy of geometry-based methods is high; however, high false negatives may occur because partially closed shallow vesicle lumens and hydrophilic cavities may not be detected, which can better bind to hydrophilic ligands. Moreover, only the vesicle lumen size is considered (excluding other factors related to ligand binding energy); thus, despite a high ligand binding energy, binding sites with unrecognized vesicle lumens cannot be identified. In addition, these methods work well in finding rigid binding pockets but are poor in predicting flexible binding pockets [38].

Energy-based methods search for pockets by calculating the interaction energy of protein atoms with small-molecule ligands (called probes); protein regions that interact well with the probe molecules are potential ligand-binding sites. For example, prediction by SITEHOUND [39] and FTSite [40] depends on the binding force between probes and binding sites, and the final ranking is based on the level of the interaction energy [39,40]. In contrast to geometry-based methods, energy-based methods do not depend on proxy indicators of ligand-binding propensity, such as cavity depth, pocket volume, or the ability to bind nonpolar spheres [39]. Although energy-based methods address issues related to the ligand binding energy, sites with vesicle-binding characteristics difficultly recognized by probes are easily overlooked and may result in partial false negatives as no quantitative assessment of protein geometric characteristics (pocket size, depth, hydrophilicity, etc.) is performed.

##### Consensus-Based

Consensus-based methods can employ algorithms that combine sequence-based, template-based, and pocket-based methods for binding site prediction. For example, MetaPocket [41] clusters the top three binding sites from sequence-based, template-based, and geometry-based predictors. The cluster with the highest total score is obtained, and the energy-based approach for residue mapping is applied to identify binding residues within the corresponding binding sites [29,42]. The purpose of using multiple algorithms in consensus methods is to address the limitations of various methods. For example, template-based methods can focus on the current target and database information for the identification of similar binding sites and improve the prediction accuracy. The addition of geometry-based and energy-based methods can provide information related to the geometric characteristics of unknown targets and ligand binding energy, which may lead to the identification of novel binding sites and compensate for the limitations of both template-based and pocket-based methods [29]. However, when using consensus methods for large amounts of data, the excessive computational length due to the addition of multiple methods and the increased computational effort are issues that need to be addressed [43].

#### 2.1.3. Machine Learning

Machine learning methods may further improve prediction accuracy by integrating the prediction information from other methods (structure-based, template-based, etc.) through machine learning algorithms. Machine learning can capture complex patterns hidden in large amounts of experimental data by building appropriate statistical models and making decisions based on them. Therefore, machine learning methods are particularly suitable for fields such as bioinformatics where large amounts of data are available but the corresponding theory is poorly developed [44]. However, as machine learning algorithms are trained on input data, they may lack data when there is limited information about the biological target of interest and thus may not produce better results [45]. The accuracy of various machine learning methods has not been compared, and there are no specific applications; thus, this review will not explore machine learning methods in detail. Further information on machine learning methods can be found in the reviews of Zhao et al. [46] and Dhakal et al. [45], who have explained the methods of machine learning and deep learning in depth.

### 2.2. Summary of Binding Site Identification Methods

The principles, related tools, applicable conditions, advantages, and disadvantages of the binding site identification methods are summarized in Table 1.

**Figure 5:**
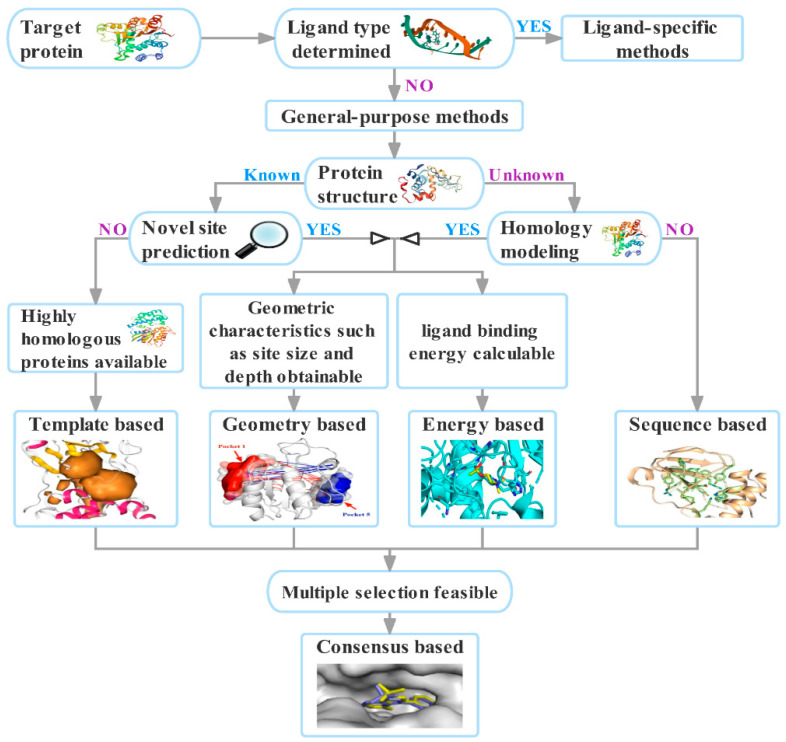
Process for choosing appropriate binding site identification methods.

Figure 5 shows the process of selecting the binding site identification method according to the target protein characteristics. Ligand-specific methods may be selected when a specific type of ligand is known. If there is no 3D protein structure, we may choose a sequence-based method. If the 3D structure of a protein is known or obtained from homology modeling, we may choose a template-based method to predict the existing binding site on the target protein. If novel binding sites need to be predicted, we may choose geometric-based, energy-based, and consensus-based methods according to the requirements. Geometric-based methods are used when geometric factors are considered, such as the size, depth, and hydrophilicity of the binding site. Energy-based methods are used when the ligand type and ligand binding energy are considered. In cases requiring a combination of these methods, consensus-based methods can be used.

### 2.3. Assessment of Druggability

Druggability evaluation is a vital step in target identification after binding site identification. Druggability is the ability of proteins to bind to drug-like molecules [48]. These interactions depend strongly on the space structure and physicochemical characteristics of the proteins and small drug-like molecules. The assessment of druggability is performed by searching for binding sites that can complement the drug-like properties of small molecules in terms of physicochemical properties [49]. Earlier druggability predictions mainly relied on experimental methods such as NMR-based fragment screening [50]; however, these methods have high costs and long cycles. These limitations may be resolved by computer-based methods, which can be used to determine the physicochemical characteristics of proteins by computational means and perform druggability analysis based on the available information without excessive experimental costs and time. Before druggability assessment, it is necessary to build a druggability prediction model to characterize protein binding sites and extract binding site descriptors to obtain scores for the protein binding sites [51]. Currently, computational druggability assessment methods can be classified as knowledge-based, sequence-based, structure-based, and hotspot-based methods.

#### 2.3.1. Knowledge-Based

The knowledge-based approach to evaluate druggability is based on known targets and ligands. Target information can be obtained in a database that includes proven druggable targets, and ligand information can be queried in the corresponding database [52]. When the proteins of interest are closely related homologs, particularly with a highly conserved sequence (e.g., >70% sequence identity), they tend to bind to chemically related ligands and catalyze similar reactions. Knowledge of the ligand/inhibitor of the related family member is likely to be useful for the design of the ligand/inhibitor of the target protein [53]. Reliable information on the target and ligand in terms of druggability can be obtained when information on homologs or family members of known targets and ligands can be found in databases, especially when these proteins have been subjected to clinical trials [52]. At present, the knowledge-based approach can be used to retrieve druggability-associated information to obtain druggability scores for target proteins, which is more accurate than other approaches for druggability evaluation [54].

#### 2.3.2. Sequence-Based

Amino acids are generally considered as key determinants of protein function. Sequence-based methods assess druggability by calculating the sequence identity between known therapeutic targets and queried target proteins utilizing machine learning or a linear regression algorithm [52]. In addition, other characteristics such as polarity, hydrophilicity, and hydrophobicity can be included in the assessment to improve predictive ability [55]. However, although the sequence-based approach helps to predict the functional domains of target proteins with target sequence information, target sequence analysis alone is not sufficient to provide concrete information about the overall structure and function of the protein target and drug–molecule interactions [55]. With sequence-based methods, only less than 10% of the human proteome is predicted to be druggable, which suggests a low accuracy [56]. In view of the accuracy and applicable conditions, these methods are often used only when the amino acid sequence of target proteins is available.

#### 2.3.3. Structure-Based

For structure-based methods in druggability assessment, the physicochemical characteristics of binding pockets are determined, which are compared with druggability pockets that have been validated [49,52]. Among the various physicochemical characteristics, the size, enclosure, and hydrophobicity of the binding pocket typically have a positive effect on druggability [5]. Specifically, the size and enclosure may be used to discriminate between non-druggable and difficult sites, whereas hydrophobicity may be used to discriminate between difficult and druggable pockets [7]. Structure-based methods fully take into account the geometric features of proteins and ligands, and predictions are in high agreement with predictions from NMR screens of fragment libraries [50,57]. In the process of druggability evaluation with structure-based methods, the binding sites of target proteins need to be obtained initially by binding site similarity searching among proteins or using a software with binding site identification functions. Then, geometric descriptors are used to represent protein characteristics to build a druggability model, whose calculated values are used to evaluate druggability [5]. The predictive accuracy of the druggability model depends on the quality, size, and diversity of the dataset [5]. Insufficient extraction of protein descriptors for target proteins can lead to over-reliance on the dataset and result in erroneous site predictions, which may adversely affect subsequent druggability evaluation [5]. Therefore, the structure-based approach can be improved in terms of prediction accuracy by adding more appropriate descriptors to shape the druggability of binding pockets and by selecting reliable datasets to reduce the effect of dataset quality [5]. In comparison with sequence-based methods, structure-based methods are more commonly used because of their higher prediction rates, and they can be used when knowledge-based methods cannot be used.

#### 2.3.4. Hotspot-Based

Protein–protein interactions usually occur at hotspots on the protein surface, which contribute greatly to the binding free energy and have a high affinity for binding to drug-like molecules [58,59]. Hotspot-based methods evaluate the druggability of target proteins by analyzing the positional relationship between binding sites and hotspots [60]. Previously, hotspots were localized mainly by NMR spectroscopy [50] and X-ray crystallography [61]. Currently, the prediction results from hotspot-based methods correlate well with the prediction results from experimental NMR-based screening [50]. Hotspot-based methods are not dependent on datasets for the acquisition and scoring of binding sites and thus do not require druggability modeling [60]. However, the approach to binding site identification in hotspot-based methods does not take into account protein flexibility and thus suffer from false negative results in prediction [60]. Although this problem can be eliminated by molecular simulations, the lack of probes in molecular simulations does not guarantee predictive success in hotspot analysis. The predictive accuracy of hotspot-based methods is inferior to that of knowledge-based methods. Therefore, hotspot-based methods are used in the assessment of druggability only when knowledge-based methods are not feasible.

### 2.4. Summary of Druggability Evaluation Methods

**Figure 6:**
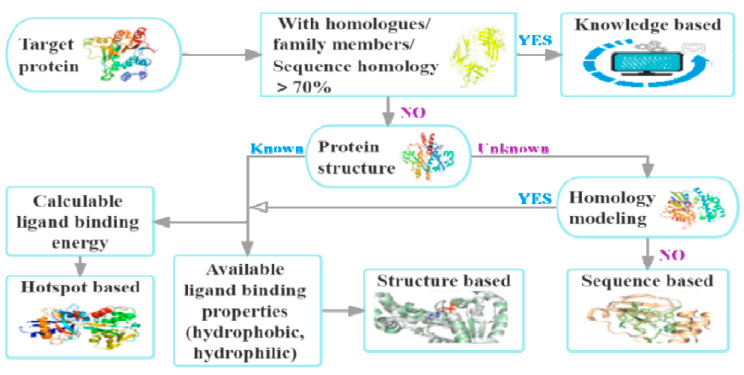
Process for choosing appropriate druggability evaluation methods.

The principles, applicable conditions, advantages, and disadvantages of methods for the assessment of druggability are summarized in Table 2. In addition, a guide to selecting methods for the assessment of druggability is presented in Figure 6. Knowledge-based methods may be selected when the database contains proteins with sufficient homologs, family members, or highly conserved sequences. When the amino acid sequence is known and there is no homology modeling structure, we may choose sequence-based methods. Structure-based or hotspot-based methods can be used when protein structures or homology modeling structures are known.

### 2.5. Differences in Binding Site Identification and Druggability Evaluation Methods

To clarify the differences between binding site identification and druggability assessment, their definition, scoring factors, relationship to each other, and purpose are summarized in Table 3.

## 3. Software and Tools

In addition to binding site identification and druggability evaluation, a good understanding of the internal software and tools of the corresponding methods is necessary to choose the appropriate tool. In general, all software and tools that can be used in druggability evaluation require information on binding sites. Therefore, they can be simultaneously used in binding site identification. These tools are introduced in two sections: (1) binding site identification and (2) binding site identification and druggability evaluation.

### 3.1. Binding Site Identification

Methods of binding site identification include ligand-specific and general-purpose methods. Various tools can be used to identify binding sites, and the commonly used web servers (or software) [16,17,18,19,20,21,22,23,24,25,26,47,62,63,64,65,66,67,68,69,70,71,72,73,74,75,76,77,78,79,80,81] of ligand-specific methods and corresponding ligand types are listed in Table S1. Table S2 shows the binding site prediction web servers (or software) [6,7,29,30,34,35,39,40,42,43,82,83,84,85,86,87,88,89,90,91,92,93,94,95,96,97,98,99,100,101,102] that apply general-purpose methods. In these tables, FOSS indicates free and open-source, and users can select the appropriate servers for binding site prediction.

In addition to these tools, a software package known as Biopython provides an online repository of modules, scripts, and links to websites for some Python-based software [103]. It is a freely available open-source tool with modules for several online databases, such as NCBI, ExPASy, SCOP, and KEGG, and is widely used in data acquisition for binding site identification and druggability assessment [103]. Notably, the provided scripts have been applied to executable visualization scripts for Pymol in Fpocket software and the construction of position-specific scoring matrices for PSI-BLAST in the COACH web server [103].

Among the tools presented in Tables S1 and S2, MetaPocket2.0 [42] and COACH are two commonly used tools in consensus-based methods, which perform well and do not have specific requirements for target proteins when only the identification of binding sites is required.

#### 3.1.1. MetaPocket 2.0

MetaPocket 1.0 [41] is a consensus algorithm consisting of four algorithms: LIGSITEcs/c [104], PASS [105], Q-SiteFinder [106], and SURFNET [107]. On the other hand, MetaPocket 2.0 [42], a free tool, added four methods: Fpocket, ConCavity, GHECOM [108], and POCASA [109]. These eight methods include sequence-based, energy-based, and geometry-based methods to further improve the predictive success rate through consensus prediction. The use of the tool consists of six steps [42]: (1) the PDB file of the protein is input into the eight algorithms, which will output various clusters of grid points and probes. The mass center of these clusters is considered as the binding sites; (2) the top three binding sites are selected from each predictor by comparing the z-score of each site calculated separately; (3) 24 sites are clustered based on spatial similarity using a hierarchical clustering method, and the center of mass of each final cluster is used as the location of the binding pockets; (4) the total z-score of each cluster is computed as the final score, which determines the confidence level of the binding sites; (5) the probe points from each method are merged in the same meta-pocket site to obtain the total meta-pocket site; (6) the NACCESS program calculates the accessibility of ligand-binding residues around the meta-pocket site. Its output includes the binding sites acquired by a single method and clustering as well as the potential active site residues for each pocket.

#### 3.1.2. COACH

COACH [29] is a consensus algorithm consisting of five algorithms (TM-SITE, S-SITE, COFACTOR [34], FINDSITE [110], and ConCavity), which include sequence-based, geometric-based, and template-based methods to further improve prediction success through consensus prediction. COACH, a free and open-source tool, is a web server developed by Extreme Science and Engineering Discovery Environment. The C-score (confidence score) of the identified binding sites is calculated as a benchmark for active site prediction. Its input is a PDB format (3D structure) or a FASTA format (2D sequence) file of the protein. A homology modeling structure is obtained using its plug-in tool (I-TASSER) [111]. Then, binding sites are identified from the template by analyzing the structural and sequence similarities (global and local) using the template-based TM-SITE method. The most consistent binding residues were obtained by sequence mapping-graph comparison using another template-based method (S-SITE) [29]. The ligand binding site prediction results of TM-SITE and S-SITE will be used with COFACTOR, FINDSITE, and ConCavity to create a prediction model by SVM and determine the binding site score as the Matthews correlation coefficient (MCC), i.e., the C-score [29]. The output of COACH includes the binding pockets with their C-scores, which indicate the accuracy and coverage of the prediction with values ranging from 0 to 1. A higher score denotes more credible pocket predictions.

### 3.2. Binding Site Identification and Druggability Evaluation

For target identification or validation, druggability evaluation after binding site identification is important; thus, tools that can be simultaneously used in binding site identification and druggability evaluation are convenient. Various tools can be used to identify the binding site and assess druggability. Table S3 lists the software [7,112,113,114,115] used for binding site identification and druggability assessment, including the pocket search method, evaluation criteria, and website link. Among these tools, PockDrug [112], FTMap [60,116], and Sitemap [7,37] are the most well-known, which can be used for both binding site identification and druggability evaluation.

#### 3.2.1. PockDrug

PockDrug is a geometry-based web server for the identification of binding sites, which offers consistent druggability results with different pocket assessment methods [112]. It can accept two types of input, one of which is the protein structure; the Fpocke or/and prox 2 methods in PockDrug will use the protein structure for binding site prediction and obtain information on the binding pocket for druggability score calculation [112]. For the other type of input, the binding pocket information is used directly for druggability score calculation [112]. Both submission methods can be used to obtain druggability scores through PockDrug prediction, which consists of seven linear discriminant analysis models with nine druggability descriptors. When the input is binding pockets, it will output the results in one table displaying pockets with at least 14 residues and a second table listing smaller pockets (<14 residues) [112]. The average druggability probability and associated standard deviation are shown, and pockets with an average druggability probability > 0.5 are considered as druggable [112]. When the protein structure is entered, the output includes the number of assessed pockets for each method, druggable pockets, and highest druggability probability with standard deviation. In either of the output forms, druggable and less druggable pockets have been reported to have average scores of 0.87 ± 0.15 and 0.18 ± 0.15, respectively [112].

#### 3.2.2. FTMap

FTMap is a web server for binding site identification and druggability scoring, whose input requires a structure file in PDB format or PDB ID. FTSite is based on FTMap, which adopts an energy-based approach when used for binding site identification [60,116]. FTMap also utilizes molecular docking as the main tool to recognize and characterize protein binding sites via a hotspot-based approach, which uses empirical energy functions and CHARMM force fields to identify possible binding sites by screening small compounds with different shapes, polarities, and sizes [60,116]. The tool aims to minimize and recalculate the free energy of the docking conformation of each probe by using 16 organic molecules as probes distributed on the surface of the macromolecule [60,116]. By clustering the sites of the 16 probes, the overlapping regions of different probe clusters are termed consensus sites (CSs). The site with the highest number of probe clusters is defined as the primary hotspot, and all other CSs are considered as secondary hotspots. The consensus cluster strength (S) is calculated as the number of probe clusters within the consensus cluster [60,116]. The output data include a PDB file, a PyMOL session file, a nonbonded interactions file, an H-bond interactions file, and a probe summary file. A consensus cluster of S > 16 and the presence of at least one additional hotspot within 8 Å of a strong hotspot indicate a druggable binding pocket [60,116].

#### 3.2.3. Sitemap

The Sitemap module in Schrödinger software can be used for binding site identification and druggability evaluation and is currently the most commonly used software. The input file of the module is a PDB file of the 3D structure of the protein, and the algorithm calculates the site score based on how close the site points are to the protein surface and how well sheltered they are from the solvent [7,37]. A series of physical descriptors including the site size, degree of enclosure, hydrophobicity and hydrophilicity of binding sites, hydrophobic/hydrophilic balance, degree of exposure to the solvent, and degree to which a ligand can donate or accept hydrogen bonds are used in the calculation of the SiteScore [7,37]. SiteScore > 0.8 can discriminate potential ligand-binding sites from all calculated sites, which allows the estimation of how tightly a novel protein target binds to ligands [7,37]. Sitemap calculates three factors affecting druggability through the identified binding sites, including the number of site points found for the sites and the degree of enclosure and hydrophilic weighting, all of which are defined as a quantitative assessment of protein druggability (Dscore) [7,37]. Its output includes SiteScore and Dscore, and the site with SiteScore > 0.8 is called the binding site; a Dscore greater than 0.83 indicates a druggable site, whereas a Dscore lower than 0.83 indicates a non-druggable binding site [7,37].

## 4. Databases

After choosing the software and tool, we must extract the target protein information from a database. Due to the expanding information on binding sites and druggable sites, there is an increasing wealth of information on known protein binding sites in databases. We identified databases containing amino acid sequences, protein structures, binding sites, druggable sites, and probe-related information in PubMed and categorized them into resource databases, probe databases, and benchmark datasets for method testing.

### 4.1. Resource Database

In target identification, we first need to determine whether a target protein has an identified binding site or a druggable site that has been clinically tested or validated; thus, a resource database is important for site prediction and druggability evaluation. The description, coverage, database type, information type, and website link of resource databases are presented in Table 4, which can be used to search for the binding sites and druggable sites of target proteins. The knowledge-based approach relies on the information on target proteins and ligands in databases for druggability evaluation. Information on target proteins or ligands can be transferred to databases to obtain binding and druggable site information. Depending on the obtained information, resource databases can be divided into three categories: sequence databases, structure databases, and drug databases. The extraction of amino acid sequences from sequence databases is the first step in target identification, and the sequence databases include UniProt [117], Research Collaboratory for Structural Bioinformatics Protein Data Bank (PDB) [118], Swiss-Model [119], and National Center for Biotechnology Information (NCBI) [120]. Structure databases are used to determine whether a target has a known structure and predicted or validated binding sites, which include PDB, Swiss-Model, NCBI, and BioLiP [121]. The Swiss-Model database is the main tool for homology modeling when the 3D structure of a target is not available. Drug databases provide information on whether a target has a druggable site that has been validated or is in clinical trials, which include DrugBank [122], Clinicaltrials.gov [123], DrugCentral [124], and PubChem [125].

### 4.2. Probe Database

In some energy-based methods, probes are required for site and hotspot prediction; thus, the type of probe should be selected from a database. The description, coverage, probe type, and website link of the available probe databases are summarized in Table 5.

### 4.3. Benchmark Datasets

A validation dataset as the benchmark is required when comparing the prediction accuracy of different servers or software. Table 6 shows benchmark datasets with different tools for the identification of binding sites. Among them, the Huang and Schroeder dataset is the most commonly used and well-known dataset in terms of validation capability [104]. In addition, structural flexibility is taken into account, and various classical binding site prediction methods use this dataset as a validation dataset; when this dataset is used, the predictive power of the new method and the classical method can be compared.

## 5. Application

After choosing a suitable tool for the corresponding method and extracting protein information from the database, the tool may be applied to the study of interest. In this review, we searched for published studies that used binding site identification and druggability evaluation methods in 2016–2022 in PubMed. In this section, we describe the applications of specific tools. First, binding site identification and binding site selection using MetaPocket and Coach are presented as examples. Then, binding site identification and further druggability assessment of the binding sites using PockDrug, FTMap, and Sitemap as examples are described.

### 5.1. Binding Site Identification

The purpose of most binding site predictions is usually to provide targets for drug design, which may play a role in influencing biological pathways. Servers for the identification of binding sites can be found in Table S2. Specifically, the use of the MetaPocket and COACH servers for site prediction is reviewed in this section.

Nanyu Han et al. [147] used the influenza neuraminidase structure snapshots from molecular dynamics simulation trajectories to identify a new motif (340-cavity) and predicted seven binding sites using a MetaPocket server. Then, they used the ligands from the ChemBridge Fragment Library for molecule docking and confirmed the new motif based on the best docking pose. Therefore, the 340-cavity may function as a novel binding pocket for designing anti-influenza drugs. 

Achintya et al. [148] obtained the *P. falciparum* DXP synthase structure by homology modeling. The consensus-based COACH approach was used to discover the Mg^2+^-binding pocket (C-score = 0.37) and ThDP-binding pocket (C-score = 0.26). Virtual screening and molecular docking of the ThDP-binding pocket were performed to screen for potential binding candidates. Ten compounds were screened as potential candidates for antimalarial drug design.

Gennaro et al. [149] obtained the protein structure models of three enzymes (monogalactosyldiacyl, sulfoquinovosyldiacyl-1, and sulfoquinovosyldiacyl-2) by homology modeling, Then, the COACH server was used to identify the UDP-binding pocket (C-score = 0.32), NAD-binding pocket (C-score = 0.85), and N-acetylglucosamine-binding pocket (C-score = 0.33). The binding sites predicted for the three synthases were consistent with those of template proteins, and the NAD-binding pocket, which had a higher C-score, could be used for drug design.

Additional examples of binding site identification performed from 2016 to 2022 are presented in Table 7, which include the databases, modeling tools or software, application methods, and prediction results. These studies demonstrate the application and value of several databases and tools in binding site identification.

### 5.2. Binding Site Identification and Druggability Assessment

The primary purpose of druggability evaluation is to screen for druggable targets through a computerized approach with the identification of binding sites, which can involve the use of the software and servers presented in Table S3. The most commonly used tools (PockDrug server, FTMap server, and Sitemap software) for binding site identification and druggability evaluation are reviewed in this section.

Froes et al. [53] obtained the 3D structure of the LasI protein from PDB. The PockDrug server was used to predict a pocket with 14 residues (P0 volume = 1306.24 Å^3^) and a pocket with 10–14 residues (P1 volume = 462.13 Å^3^); P0 (druggability score = 1.0) and P1 (druggability score = 0.92 ± 0.05) were further considered druggable. In addition, the first hotspot (S = 24) in the FTmap’s hotspot assay was located at a distance of 7.05 Å (center-to-center distance) from the second hotspot and met the druggability criteria. P0 and P1 could be used to design drug inhibitors against the bacterial resistance pathway.

Du et al. [150] obtained the structure of the OSM-OSMR complex by homology modeling and submitted it to the FTMap web server, resulting in the detection of ten potential drug-binding sites and eight hotspots in the OSM-OSMR complex, six of which (sites 0, 1, 3–6) were at the interface of the OSM-OSMR interaction. The eight hotspots were mapped to protein residues to evaluate the druggability of the predicted binding sites in the OSM-OSMR complex. Among these hotspots, there were two hotspots respectively at sites 1, 3, and 6, one hotspot at both sites 0 and 4, one hotspot at site 5, and no hotspots at sites 2 and 7–9. Therefore, sites 1, 3, and 6, which have the most hotspots, are important druggable sites for designing inhibitors that may inhibit the OSM-OSMR interaction.

Ruchi et al. [151] obtained the protein structure of CHRNA7 from *Homo sapiens* (humans) by homology modeling, submitted it to the Sitemap software, and identified five binding sites. There were four binding sites with both SiteScore > 0.8 and Dscore > 0.83, and one site had a SiteScore < 0.8. Therefore, only the four binding sites are druggable and may be used to design inhibitors of the CHARNA7-related biological pathway.

In contrast, Adeniji [152] obtained the dynamic structure of the K-RasG12C variant from molecular dynamics simulations, and six binding sites were consistently predicted using the Sitemap, SiteHound, and MetaPocket 2.0 servers. Four of the six binding sites were predicted as druggable with a SiteScore > 0.8 and a Dscore > 0.83. The binding sites were evaluated according to their size, burial, and hydrophobicity scores; site 2 exhibited relatively higher hydrophobicity and hydrogen donor/acceptor properties, which may be used to design next-generation K-Ras inhibitors.

Additional examples of binding site prediction and druggability evaluation performed from 2016 to 2022 are presented in Table 8, which include the databases, modeling and evaluation tools, binding site identification tools, application methods, and prediction results. These studies provide in-depth information on the process of druggability evaluation.

**Table 7:** Applications of databases/tools in binding site identification from 2016 to 2022.

Year	Database	Modeling Tool/Software	Tool for Model Quality Assessment	Tool	Prediction Result	Reference
2016	PDB	Swiss-Model [153]	QMEAN [154], PROCHECK [155], ProSA [156], Verify3D [157]	Fpocket	4 binding pockets	[158]
2016	PDB	GROMACS program suite		MetaPocket	7 binding sites	[147]
2016	PDB	Phenix [159]	MolProbity [160]	MetaPocket 2.0	3 binding pockets	[161]
2016	UniProtKB [162], PDB	Molecular Operating Environment		Site Finder	3 binding pockets	[163]
2016	UniProt	Modeller [164]	PROCHECK, ProSA, Swiss-PDB Viewer [165]	CASTp [36], Q-SiteFinder, Sitemap	CASTp: 2 binding cavities, 11 binding residues; Q-SiteFinder: 2 binding cavities, 11 binding residues; R-Sitemap: 1 binding site region, 7 binding residues	[165]
2017	PDB	HHPred [166], RaptorX [167], (PS)2 server [168], Modeller	RAMPAGE [169], QMEAN	COACH	2 binding sites, 17 binding residues	[148]
2017	PDB	Modeller	SAVES [170], ProSA	Sitemap	13 binding residues	[171]
2017	NCBI, PDB	NAMD	——	FTMap	5 binding sites, 41 binding residues	[172]
2017	NCBI	I-TASSER	PROCHECK, ProSA, QMEANclust [173]	COACH	1 binding site, 18 binding residues	[174]
2018	Uniprot	Swiss-Model, PRIME module of Schrödinger	ProtParam [175], PROCHECK	Sitemap	4 binding sites	[176]
2019	PDB	Modeller	PROCHECK, ProSA	Sitemap	4 binding cavities	[177]
2019	UniProt	Modeller	SAVES, PROCHECK, Verify3D	Sitemap	1 binding pocket, 19 binding residues	[178]
2019	PDB	——	——	FTSite	18, 29, and 40 binding residues on 3 proteins	[179]
2020	UniProt, PDB	Swiss-Model	TM-align server [180]	LISE, Sitemap	1 consensus binding site	[181]
2020	PDB, UniProt	Modeller	ProSA, Verify3D	CPORT [182], Sitemap	1 consensus binding site, 38 binding residues	[183]
2020	Uniprot, PDB	Modeller	PROCHECK, Verify3D, ProSA	CASTp, Sitemap, PatchDock [184]	CASTp: 10 binding residues Sitemap: 16 binding residues PatchDock: 3 binding residues	[185]
2020	PDB	PHYRE2 software [186]	PSVS server, PROCHECK, Verify3D, ProSA	Sitemap	90 binding residues	[187]
2021	PDB, UniProt, GenBank, Pharos, PubChem	Swiss-Model	PROCHECK, ProSA, ProQ, Verify3D, PROVE, ERRAT [188]	DoGSite	3 binding pockets	[189]

## 6. Discussion

### 6.1. Comparison of Tools for Identification of Potential Drug Targets

As part of the process of target identification, it is necessary to choose a well-known and appropriate tool for binding site identification and druggability evaluation. To compare the applications of various tools in binding site identification and druggability assessment, we counted the number of studies that used geometric-based, energy-based, and consensus-based methods (Table 7). However, we did not include studies that used template-based and sequence-based methods because their accuracy in binding site prediction is inferior to the three aforementioned methods, and no relevant articles have been published in the last six years. Based on Table 7, geometry-based methods are more widely used than energy-based and consensus-based methods in binding site identification. In addition, the Sitemap module of Schrödinger software was used in 17 studies (Table 7), which was the most widely used tool among all tools. Table 8 shows the applications of various druggability evaluation methods, including Sitemap, PockDrug, Fpocket, and FTMap, from 2016 to 2022, and Sitemap was the most widely used evaluation method, accounting for 53.3% of the total studies. The findings suggest that most researchers focus on geometric characteristics by using Sitemap in binding site prediction, which allows ligand design with high accuracy. Therefore, Sitemap is currently the main target identification software, and its usage rate may increase in the future.

In the process of target identification, both binding site identification and druggability evaluation are necessary. In this review, tools that can accomplish both functions simultaneously (PockDrug, FTMap, and Sitemap) are compared with a focus on their advantages.

#### 6.1.1. PockDrug

The PockDrug server uses a linear discriminant analysis approach with 52 geometrical and physicochemical descriptors and calculates the average druggability probability to make a consensus forecast of pocket druggability [112]. The advantages of this tool are as follows:

(1) It provides both the average druggability probability and its corresponding standard deviation [112];
(2) The server accepts any structures, including X-ray, NMR, homology, or docking structures, in PDB format as input [112];
(3) The PockDrug model can be used to directly score the druggability of pockets based on the results of pocket estimation methods, and, importantly, it is valid for different pocket estimation methods [112];
(4) A comparison of PockDrug, Fpocket, and DoGSite in terms of the prediction sensitivity, accuracy, and MCC suggests that PockDrug performs better than Fpocket and DoGSite [112].

#### 6.1.2. FTMap

FTMap is a tool for energy-based site prediction and druggability evaluation, which plays a major role in target identification. The advantages of the FTMap method are as follows:

(1) The computerized results of FTMap are consistent with the experimental results of NMR-based screening, demonstrating the accuracy of hotspot prediction [60];
(2) The probe types used in FTMap can accurately identify binding sites and provide the robustness required to eliminate false positives (e.g., sites within narrow lumens) [116];
(3) The use of a detailed energy expression profile to locate probes on the surface of sampled proteins and the Fourier transform correlation approach ensures its high accuracy [60];
(4) The method does not need a training dataset and thus does not depend on the quality, size, and diversity of the benchmark and validation datasets, which can minimize the potential effect of pocket predictions with different accuracies on the subsequent evaluation of druggability;
(5) The method can be employed for all types of protein structures for site prediction without prior knowledge of similar structures or potential binding sites [116].

#### 6.1.3. Sitemap

In comparison with other methods, the Sitemap method has the following advantages:

(1) The performance of Sitemap for large-scale validation/test datasets is excellent with 86% and 96% accuracy, which is higher than that of Fpocket, DoGSiteScorer, and PockDrug [37];
(2) Sitemap provides quantitative and graphical information about the active site, which can help guide the modification of the ligand structure. In particular, its interface can be divided into hydrophilic, hydrophobic, and neither hydrophilic nor hydrophobic regions [37]. As an example, this can help us to determine whether there is space to accommodate hydrophobic regions with larger hydrophobic groups to help design better ligands with stronger binding affinity. Modifying the ligand’s physical properties to improve potency can facilitate the subsequent molecular docking or virtual screening in drug design [37];
(3) The structures of most proteins used in drug prediction are currently unknown; thus, homology modeling is required. The Prime module of the Schrödinger software package allows homology modeling, providing convenience through the use of the same software [37];
(4) Prediction by Sitemap is more accurate for enzyme sites than for receptor sites [7].

### 6.2. Comparison of PockDrug, FTMap, and Sitemap

Given the advantages and disadvantages, how do we choose PockDrug, FTMap, or Sitemap? First, geometry-based methods are preferred because the size and shape of binding sites are the key factors in determining their binding affinity. Although ligand binding energy is also important for prediction, geometry-based methods can predict most of the binding sites. However, the energy-based FTMap tool excludes geometric features, resulting in a slightly less accurate prediction compared with a geometry-based tool. In addition, FTMap does not take into account protein flexibility. Although this shortcoming can be addressed using molecular dynamics simulations, current hybrid molecular dynamics approaches rely on only a few probe types, which may limit the reliability of hotspot prediction [116]. Moreover, another limitation of FTmap is that the analysis of proteins with more than 1100 residues often fails due to the memory limitations of computational resources. Therefore, the FTMap method should not be chosen for the prediction of large-molecule proteins [116]. Despite the acceptable accuracy of FTMap in druggability prediction, Sitemap and PockDrug (geometry-based methods) are often the first choice owing to high prediction rates and fewer restrictions. Furthermore, a comparison of Sitemap and PockDrug has shown that the accuracy of Sitemap and its supporting role in ligand design are more helpful for drug design. Therefore, we recommend Sitemap as a primary method for binding site identification and druggability evaluation.

### 6.3. Recommended Methods for Identification of Potential Target Binding Sites

Although Sitemap may be a primary method for the prediction of binding sites and druggability, the use of two different types of methods simultaneously is preferable for consistent prediction. The consistent prediction of target binding sites by two types of methods can identify additional features and address the limitations of the respective methods. For example, the classical server, COACH, combines sequence-based, geometric-based, and template-based methods for consensus prediction, which performs better than any of the methods alone, demonstrating the effectiveness of a combination of methods [29]. As shown in Table S3, a total of five methods could be chosen. Therefore, taking into account the tool type, accuracy, advantages, disadvantages, and frequency of application, we recommend using either Sitemap or PockDrug in conjunction with FTMap for the consistent prediction of binding sites and druggability. Sitemap and PockDrug are geometry-based methods, and FTMap is an energy-based method. Froes et al. [53] applied the PockDrug and FTMap methods to the Mvf protein and consistently predicted two binding pockets with satisfactory results for both methods. They found that 3-amino-7-chloro-2-nonylquinazolin-4(3H)-one and the benzimidazole derivative M6443 could bind to both pockets on the MvfR protein, demonstrating that a combination of PockDrug and FTmap could address the limitations of the two methods and improve the accuracy of druggability evaluation [53]. The druggable sites consistently predicted by both methods were more druggable than those predicted by only a single method due to their excellent combined performance in terms of geometric features and ligand binding energy. In the case of inconsistently predicted druggable sites by PockDrug and FTMap, they may be considered as alternative drug targets instead of non-druggable sites, which may be used as additional sites in multi-target drug design. From a theoretical perspective, the greater the number of methods, the greater the number of alternative target sites. However, the use of a combination of closely similar methods is not recommended because in cases involving highly similar features, the predicted results are often similar. Moreover, multi-method applications prolong the computational cycle and time, which would hardly contribute to an improvement in accuracy.

### 6.4. Previous Reviews of Binding Site Identification and Druggability Assessment

To help readers achieve a better understanding of binding site identification and druggability assessment, we searched the literature published in the last five years in PubMed and identified five articles closely related to this review. Three of them are about the principles, applications, drawbacks, and improvement of methods used in the identification of binding sites, and the other two articles describe the principles, software, databases, and inadequacy of current methods in the assessment of druggability.

#### 6.4.1. Binding Site Identification

Neal and Mahmoud [205] briefly summarized and categorized the methods used in the identification of binding sites and assessment of druggability. They described the principles underlying each method and provided information on some software and web server sites. The limitations of the current methods in the identification of binding sites and assessment of druggability are highlighted, which revealed that the dynamic binding of protein ligands and the chemical environment are usually not taken into account [205]. It is suggested that protein–ligand binding sites and binding modes obtained by molecular docking should be used as the basis for binding site identification and druggability evaluation [205]. Then, the binding free energy and entropy can be calculated by accelerated molecular dynamics simulations and binding thermodynamics to verify the stability of the binding sites in the docking results and to account for the dynamics of protein–ligand binding and the chemical environment [205].

Feng and Khaled [206] briefly described the classification and principle of binding site identification, introduced FTMap in energy-based binding site identification, stated the evaluation criteria of druggability evaluation, and introduced Dscore and FTMap to calculate druggability. They also used HIV integrase and Ras proteins as examples to demonstrate the importance of considering protein flexibility and recommended the use of cosolvent molecular dynamics simulation to take into account protein flexibility in order to obtain variable binding sites that are difficult to identify with current binding site identification methods and increase the number of alternative targets [206]. However, this method is limited by a low solvent diffusion rate and the restricted use of probes.

Zhao et al. [46] systematically introduced sequence-based, template-based, structure-based, and machine learning algorithms for binding site prediction. In particular, the review highlights recent advances in deep learning methods and compares them with traditional machine learning algorithms [46], showing that deep learning outperforms traditional machine learning in many data processing aspects [207,208,209,210]. However, more expensive computational costs and resources are needed. The authors also point out that the current range of methods for predicting binding sites has some limitations and does not fully address existing issues. Therefore, potential binding sites may be identified by combining protein conformational sampling (e.g., molecular dynamics simulations), which could be a new direction for future research [46].

#### 6.4.2. Druggability Assessment

Sarah et al. [211] briefly described the principles of ligand-based and structure-based druggability evaluation methods, listed the common databases of commonly used ligand-based methods, and elucidated structure-based druggability evaluation methods. In addition, the rationales and differences of network-based, ligand-based, and structure-based methods are summarized. The network-trained mechanistic models of network-based methods identify drug targets and analyze potential and existing drugs through molecular interaction relationships [211].

Clement et al. [212] described the principles of druggability, examined drug similarities, and analyzed the reliability of different computational tools for prediction in the field of drug discovery. Druggability evaluation methods were classified as sequence-based, structure-based, ligand-based, and precedent-based. Specifically, the principles, reliability, common computational tools, and shortcomings of these methods were described, and the disadvantages of the druggability models built by these methods were pointed out [212]. First, the accuracy of target prediction based on known protein structures was demonstrated to be only 70%. Second, the available information on the druggability models obtained using these methods was mainly the geometric features of proteins, and protein–ligand interactions were usually excluded. Therefore, integrating information on druggability and the lead compound’s drug-likeness may achieve better prediction results.

#### 6.4.3. Comparison between Previous Reviews and This Review

In Table 9, we summarize the questions or issues posed with the corresponding solutions of the previous five reviews and our review. In comparison with the previous reviews, our review not only briefly describes the current common methods, principle, databases, and software for binding site identification and druggability evaluation but also presents the current applications of commonly used methods in the last six years and compared these methods and their applications in the discussion section. Specifically, we compared the features of PockDrug, FTMap, and Sitemap and identified Sitemap as the preferred method for protein binding site identification and druggability evaluation. Furthermore, instead of using the binding site and druggability evaluation methods individually, we suggest combining Sitemap or PockDrug with FTMap for consistent prediction, which could address the limitations of each method and allow the consistent prediction of target binding sites.

### 6.5. Potential and Improvement of Methods for Identification of Potential Drug Targets

Despite the use of traditional experimental approaches in drug discovery over several decades, the overall failure rate of drug development is still around 96% due to the ‘unpredictability’ of disease targets [212]. Incorrect and inappropriate target selection is a key contributor to the high cost and low effectiveness of current drug discovery programs [213]. Computer-aided drug design methods have emerged due to the need to reduce expenses, experimental time, and research effort. With the availability of a large number of X-ray structures, abundant genomic data, and updated methods for binding site and druggability prediction, it is increasingly feasible to use computer-based prediction methods instead of traditional experimental methods for biological targets. This approach can greatly reduce the number of targets applied in clinical trials at a later stage, shorten the experimental time, and reduce research costs. With the increasing number of articles on the application of computer-based methods for target identification, the use of computer-aided drug design methods for target binding site identification appears to be gaining popularity.

However, despite the numerous advantages of computer-based binding site identification and druggability evaluation, there are some noteworthy drawbacks. The 3D structures of proteins obtained from databases or homology modeling imply that these protein structures have a single static pattern instead of a flexible pattern [214]. Previous studies have highlighted the negative implications of overlooking protein flexibility [206,215,216]. Furthermore, protein flexibility is an important feature that affects ligand binding and affinity, which can have a huge impact on the molecular docking step in target identification [215,217,218]. To account for the flexibility of proteins, we may use a collection of protein conformations. However, the availability of suitable experimental structures is usually low; thus, there are insufficient conformations to reflect the flexibility of the target protein [219]. Molecular dynamics simulations could be used to obtain sufficient protein conformations through snapshots. Some studies have described the use of molecular dynamics simulations to resolve protein flexibility problems [49,220,221].

Currently, two molecular dynamics simulation methods can be used, i.e., the cosolvent molecular dynamics simulation method [222] and multiple-ligand-mapping molecular dynamics (mLMMD) method [223]. In the cosolvent molecular dynamics simulation method, protein structures are immersed in different concentrations of various solvents, and the solvent molecules act as probes by diffusion and interact with hotspots on the protein surface [224]. The location of binding sites on the protein surface is mapped by the probes, and different types of probes identify sites that favor specific interactions. In contrast to conventional binding site identification methods, the simulation method ranks the binding sites based on the occupancy time and local density of organic molecules, thus determining druggability according to the time of solvent molecules occupying the binding sites [224]. This approach eliminates issues related to protein flexibility and solvent during simulation and allows the prediction of more variable conformations and cryptic binding sites, such as the previously unknown cryptic binding pockets of the Bcl-xL protein [225]. The cosolvent simulation method uses a probe type that allows only one probe type to be used at a time, indicating that multiple simulations must be carried out to obtain different types of binding sites; on the other hand, mLMMD can use multiple types of probes at the same time and can map different types of binding sites simultaneously, thus improving efficiency [223]. In addition, the high probe concentrations used in some methods of co-solvent simulation require the introduction of artificial repulsions to prevent probe aggregation, which may lead to unexpected mapping artifacts; this issue does not occur with mLMMD [223]. The ability of mLMMD in identifying cryptic binding pockets is excellent, which has been demonstrated for three proteins with known cryptic binding pockets (IL-2, PLK1, and p38 MAPK) [223]. Overall, the mLMMD method is preferred to compensate for the protein flexibility and solvent issues of the conventional method.

## 7. Conclusions

In this review, we summarized the fundamentals of computer-based methods for potential therapeutic target identification, including binding site identification and druggability assessment, by comparing the differences of various methods and summarizing the databases and tools required for these methods. In addition, we identified and summarized representative studies related to binding site identification and druggability evaluation methods performed from 2016 to 2022. We compared the current tools available for both binding site identification and druggability evaluation, (i.e., PockDrug, FTMap, and Sitemap) and demonstrated the potential of these methods. We believe that binding site identification and druggability evaluation methods that allow consistent prediction will become increasingly popular for target identification, and more users will adopt mLMMD to account for protein flexibility. The purpose of this review is to help readers understand the steps and available computational tools of binding site identification and druggability evaluation, become familiar with the applications of these methods, and better utilize the currently available tools for target identification.

## Figures and Tables

**Table 1:** Comparison of binding site identification methods.

Method			Principle	Example Tool	Available	Applicable Conditions	Advantage	Disadvantage
Ligand-specific method			Interaction with different types of ligands	SXGBsite [47]	FOSS	Require specific ligand types	Accurate prediction of sites for the desired ligand type	Poor performance for non-specific ligand types
General-purpose methods	Sequence-based		Residue conservation	Concavity	FOSS	Only known sequence	Effective identification of sequence-conserved sites	Exclude physicochemical characteristics
	Template-based		Sequence similarity	LIBRA-WA	NA	Known protein with high homology in databases	Acceptable predictive ability for conserved sites	Poor prediction of novel sites
	Structure-based	Geometry-based	Geometric characteristics	Sitemap	FOSS	Require specific geometric features	High prediction rates in large and superficially bound cystic cavities	Do not consider ligand binding energy
		Energy-based	Energy of interactions	FTSite	Free	Require excellent ligand binding energy	Superior performance in predicting ligand binding energy	Exclude geometric features
	Consensus-based		Comprehensive assessment of the above four methods	COACH	FOSS	All feasible	Address inter-method limitations	Time consuming with huge amounts of data

Note: FOSS = free and open-source software; NA = not available.

**Table 2:** Comparison of methods for the assessment of druggability.

Method	Principle	Applicable Conditions	Advantage	Disadvantage
Knowledge-based	Data searching	Known homolog or family member	Highest prediction accuracy	Strict search requirements may lead to no results
Sequence-based	Machine learning and linear regression	Only known sequence	Easy access to data	Low prediction accuracy with lack of dynamic analysis
Structure-based	Geometric and energetic criteria on 3D grids	Known structure	Focus on geometric and energy characteristics	Dataset performance affects prediction accuracy
Hotspot-based	Geometric and energy characteristics		Based on ligand binding energy	Exclude protein flexibility and geometric features

**Table 3:** Comparison of binding site identification and druggability evaluation.

Method	Definition	Key Scoring Factor	Relationship	Purpose
Binding site identification	Selection of binding regions with good ligand binding ability	Site size, depth, burial properties, and ligand binding capacity	Provide site information for druggability evaluation	Design inhibitors and antagonists to target binding sites
Druggability assessment	Screening for binding sites with drug-like molecule binding ability	Size, enclosure, and hydrophobicity	Independent of or dependent on site prediction	Reduce the number of potential binding sites or predicted targets

**Table 4:** Resource databases for searching binding sites and druggable sites.

Database	Description	Coverage	Database Type	Information Type	Extracted Date	URL	Available
UniProt	A collection of sequences and annotations	568,002 manual annotation, 226,771,949 automated annotation	Sequence	Target	2022/4/28	http://www.uniprot.org/	√
Swiss-Model	A collection of homology modeling structures	2,260,758 models, 183,354 structures	Sequence, structure	Target	2022/8/24	http://swissmodel.expasy.org/	√
PDB	3D structural data for large biological molecules	194,820 structures, 1,000,361 computational structure models	Sequence, structure, drug	Target, ligand	2022/8/23	https://www.rcsb.org/	√
NCBI	A search and retrieval system of sequences, including structural data and images	33,664,932 genes, 968,236,913 proteins, 110,628,849 compounds	Sequence, structure, drug	Ligand	2021/9/4	https://www.ncbi.nlm.nih.gov/	√
BioLiP	A database for high-quality, biologically relevant ligand-protein binding interactions	116,643 proteins, 23,492 entries with binding affinity data	Structure	Target	2022/4/1	http://zhanglab.ccmb.med.umich.edu/BioLiP/	√
PubChem	A database with molecules such as nucleotides, carbohydrates, lipids, and peptides	111,889,485 compounds, 185,291 proteins	Structure, drug	Target, ligand	2022	https://pubchem.ncbi.nlm.nih.gov/	√
BindingDB [126]	A database of binding affinities and interactions of drug targets with small, drug-like molecules	2,588,694 binding data	Structure, drug	Target, ligand	2022/8/28	https://www.bindingdb.org/bind/index.jsp	√
PDTD [127]	A web-accessible protein database for in silico target identification	>830 known or potential drug targets	Structure, drug	Target	——	http://www.dddc.ac.cn/pdtd/	×
DrugCentral	An online drug information resource on active ingredients, chemical entities, etc.	4714 drugs, 129,975 pharmaceuticals	Drug	Target	2022/7	http://drugcentral.org/	√
Clinicaltrials.gov	A web-based resource of clinical studies on diseases and conditions	426,507 studies	Drug	Target	2022/8/23	https://clinicaltrials.gov/ct2/home/	√
DrugBank	An online database containing information on drugs and drug targets	14,755 drug entries	Drug	Target	2022/1/3	https://go.drugbank.com/	√
KEGG [128]	A database resource for high-level functions and utilities of the biological system	18,965 substances, 11,953 drugs	Drug	Target	2022/7/1	http://www.kegg.jp/	√
IUPHAR [129]	An expert-curated resource of pharmacological targets and substances	3002 targets, 11,348 ligands	Drug	Target, ligand	2022/6/9	https://www.guidetopharmacology.org/	√
ChEMBL [130]	A database of molecules with drug-like properties, chemicals, and bioactivity	15,072 targets, 2,331,700 compounds	Drug	Target	2022/7/12	https://www.ebi.ac.uk/chembl/	√
TTD [131]	A database consisting of target-interacting proteins, patented agents, and their targets	3578 targets, 38,760 drugs	Drug	Target	2021/11/8	http://db.idrblab.net/ttd/	√
SwissTargetPrediction [132]	A website to estimate the most probable macromolecular targets of a small molecule	3068 targets, 376,342 active compounds, 580,496 interactions	Drug	Ligand	2019	http://swisstargetprediction.ch/	√

**Table 5:** Probe databases for the identification of binding sites and assessment of druggability.

Database	Description	Coverage	Probe Type	Species	URL	Available
PhylOPDb [133]	A web interface to browse 16S rRNA-targeted probes	74,003 probes	Oligonucleotide	Bacteria and Archaea	http://g2im.u-clermont1.fr/phylopdb/	√
ProbeBase [134]	A database of rRNA-targeted oligonucleotide probes and primers	2788 probes, 175 PCR primers	Oligonucleotide	Microorganism	http://www.probebase.net/	√
R-BIND [135]	A database with tools for probe development and information	113 ligands	RNA	——	https://rbind.chem.duke.edu/	√
RTPrimerDB [136]	A public database of PCR primer and probe sequence records	Probe records	Nucleotide	Human, rat, mouse, fruit fly, and zebrafish	http://www.realtimeprimerdatabase.ht.st/	×

**Table 6:** Benchmark datasets with different tools for the identification of binding sites.

Dataset	Year	Coverage	Source Database	Applied Tool
Huang and Schroeder	2006	48 unbound/bound structures and 210 bound structures	PLD [137], ConSurf HSSP [138], PDB	LIGSITEcsc [104], MetaPocket, MetaPocket 2.0, FTSite, Fpocket, DoGSite [139], COFACTOR, P2Rank [43], PocketPicker [140], PUResNet [89], EXPOSITE [141], VICE [142], ISMBLab-LIG [143], MSPocket [101], bSiteFinder [31], POCASA [109]
FINDSITE	2008	901 protein–ligand complexes	PDB	FINDSITE, 3DligandSite [144], LISE
COACH validation set	2013	500 proteins, 815 binding ligands	BioLiP	COACH
LigASite dataset (v7.0)	2009	337 proteins with apo (unbound) structures	PDB, HSSP [145], Catalytic Site Atlas [146]	ConCavity
MPLs-Pred validation set	2019	234 proteins	UniProt	MPLs-Pred
Sitemap validation set	2009	538 proteins	PDB	Sitemap
COFACTOR validation set	2012	450 non-homologous proteins	PDB	COFACTOR
SXGBsite validation set	2019	5 nucleotides, 5 metal ions, DNA, and hemoglobin	BioLiP	SXGBsite

**Table 8:** Applications of databases/tools in druggability assessment from 2016 to 2022.

Year	Database	Modeling and Evaluation Tool	Binding Site Identification Tool	Web Server/Software for Druggability Assessment	Prediction of Druggability	Reference
2016	UniProtKB, Cluster, PDB	Prime module of Schrödinger software, PROCHECK	Sitemap		1 druggable binding site (Dscore = 1.33)	[190]
2017	UniProt	Prime module of Schrödinger software	Sitemap		1 druggable binding pocket (Dscore = 1.228)	[151]
2018	PDB	Swiss-Model	Sitemap		5 sites (SiteScore > 1 is druggable)	[191]
2018	PDB	Modeller, HHpred, PRIMO	SiteHound, MetaPocket 2.0, Sitemap	Sitemap	4 of 6 sites are druggable (Dscore > 0.83)	[152]
2019	PDB	PyMOL module of Schrödinger software, Schrödinger Multiple Sequence Viewer	Sitemap		1 druggable binding pocket	[192]
2019	PDB, PDBind, MOAD	——	PockDrug, FTMap		LasI protein: 6 binding sites (2 are druggable, scores of 1.0 and 0.92 ± 0.05, respectively)	[53]
2019	NCBI, UniProt, PDB	Modeller, HHpred, PRIMO, ProSA, Verify3D, QMEN	FTMap and Sitemap		Binding sites of 3 of 4 models are druggable	[193]
2020	PDB, NCBI	Blast [194], Modeller	FTMap		3 of 10 binding sites are druggable	[150]
2020	PDB	——	DoGSite, FTMap, CryptoSite	Sitemap	NUDT1, NUDT5, NUDT7, NUDT9, NUDT12, NUDT15, NUDT17, and NUDT22 are druggable	[195]
2020	NCBI, GEO, PDB	I-TASSER, Swiss-Model	Fpocket		14 genes are druggable	[196]
2021	PDB	Markov state model	TRAPP and Sitemap		All pockets (except PDB ID 6WTK) are druggable	[197]
2021	TriTrypDB, BindingDB, UniProt, PDB	Swiss-Model	Fpocket		599 (87.9%) and 629 (88.8%) protein structures with druggable binding sites	[198]
2021	TCGA [199], STRING [200]	——	PockDrug		1 of 3 predicted protein pockets is druggable	[201]
2021	PDB, UniProt, GenBank, Pharos, PubChem	Swiss-Model, Phyre2, I-TASSER, Verify3D, PROCHECK, ProQ, ERRAT, ProSA	MetaPocket 2.0, CavityPlus [202], Pocket Match [203], ConSurf	PockDrug	All 4 binding pockets > 0.91	[204]

**Table 9:** Comparison of Previous Reviews.

Reference	Year	Questions/Issues Posed	Solution
[205]	2017	Lack of protein flexibility	Molecular dynamics simulations, molecular docking and combined thermodynamic methods
[206]	2018	Lack of protein flexibility	Cosolvent molecular dynamics simulation
[46]	2020	Unable to identify mystery sites	Protein conformation sampling techniques
[211]	2017	Insufficient accuracy of druggability methods	Identify drug targets and new uses for old drugs using a web-based approach
[212]	2020	Inadequate prediction accuracy and exclusion of protein–ligand interactions	Combine druggability and drug-likenesses
This review	2022	How to improve prediction accuracy	Ensure consistent prediction

## Data Availability

Not Applicable.

## Supplementary Materials

The following supporting information can be downloaded at: https://www.mdpi.com/article/10.3390/molecules27207103/s1, Table S1: Ligand-specific methods in the identification of binding sites; Table S2: General-purpose methods in the identification of binding sites; Table S3: Software or tools in the assessment of druggability.
