# Supplementary material for: In Silico Methods for Identification of Potential Active Sites of Therapeutic Targets
Source: Molecules. 2022 Oct 20;27(20):7103. doi: 10.3390/molecules27207103 (PMC9609013; doi:10.3390/molecules27207103)
Supplement: Supplementary file 1 [file molecules-27-07103-s001.zip › molecules-1818514-supplementary.pdf]

# In Silico Methods for Identification of Potential Active Sites of Therapeutic Targets

Jianbo Liao, Qinyu Wang, Fengxu Wu and Zunnan Huang

Table S1 Ligand-specific methods in the identification of binding sites

| Names | Year | Methods | Predicted type of binding site | URL | Available |
|---|---|---|---|---|---|
| CHED [1] | 2007 | Web server | Metal-binding site | http://ligin.weizmann.ac.il/ched/ | NA |
| FINDSITE-metal [2] | 2011 | Web server | Metal-binding site | http://cssb.biology.gatech.edu/findsite-metal/ | FOSS |
| IonCom [3] | 2016 | Web server | Metal-binding site and acid radical ion-binding site | http://zhanglab.ccmb.med.umich.edu/IonCom/ | FOSS |
| ATPint [4] | 2009 | Web server | ATP -binding site | http://www.imtech.res.in/raghava/atpint/ | NA |
| ATPsite [5] | 2011 | Web server | ATP -binding site | http://biomine.ece.ualberta.ca/ATPsite/ | NA |
| TargetATPsite [6] | 2013 | Web server | ATP -binding site | http://www.csbio.sjtu.edu.cn/bioinf/TargetATPsite/ | Free |
| ATPbind [7] | 2018 | Web server | ATP-binding site | http://zhanglab.ccmb.med.umich.edu/ATPbind/ | Free |
| MetaDBSite [8] | 2011 | Web server | DNA-binding site | http://sysbio.zju.edu.cn/metadbsite/ | Free |
| DNABR [9] | 2012 | Web server | DNA-binding site | http://www.cbi.seu.edu.cn/DNABR/ | Free |
| TargetDNA [10] | 2016 | Web server | DNA-binding site | http://202.119.84.36:3079/TargetDNA/ | Free |
| DRNApred [11] | 2017 | Web server | DNA/RNA-binding site | http://biomine.cs.vcu.edu/servers/DRNApred/ | Free |
| NsitePred [12] | 2012 | Web server | Nucleotides-binding site | http://biomine.ece.ualberta.ca/nSITEpred/ | NA |
| TargetS [13] | 2013 | Web server | Nucleotides-binding site | http://www.csbio.sjtu.edu.cn/bioinf/TargetS/ | Free |
| TargetSOS [14] | 2014 | Web server | Nucleotides-binding site | http://www.csbio.sjtu.edu.cn/bioinf/TargetSOS/ | Free |
| TargetNUCs [15] | 2016 | Web server | Nucleotides-binding site | http://csbio.njust.edu.cn/bioinf/TargetNUCs/ | Free |
| SXGBsite [16] | 2019 | Software | Nucleotide-binding site，DNA-binding site，Mg2+-binding site | https://github.com/Lightness7/SXGBsite/ | FOSS |
| GraphBind [17] | 2021 | Web server | Nucleic-acid-binding site | http://www.csbio.sjtu.edu.cn/bioinf/GraphBind/ | FOSS |
| DRAF [18] | 2018 | Web server | TF-binding site | https://www.cbrc.kaust.edu.sa/DRAF/index.php/ | FOSS |
| MTTFsite [19] | 2019 | Web server | TF-binding site | http://hlt.hitsz.edu.cn/MTTFsite/ | FOSS |
| Anchor [20] | 2019 | Software | TF-binding site | https://github.com/GuanLab/Anchor/ | FOSS |
| FactorNet [21] | 2019 | Software | TF-binding site | http://github.com/ucicbcl/FactorNet/ | FOSS |
| Leopard [22] | 2021 | Software | TF-binding site | https://github.com/GuanLab/Leopard/ | FOSS |
| DeepGRN [23] | 2021 | Software | TF-binding site | https://github.com/jianlin-cheng/DeepGRN/ | FOSS |
| RBinds [24] | 2020 | Web server | RNA-binding site | http://zhaoserver.com.cn/RBinds/RBinds.html/ | Free |
| iCircRBP-DHN [25] | 2021 | Software | RNA-binding site | https://github.com/houzl3416/iCircRBP-DHN/ | FOSS |
| TargetM6A [26] | 2016 | Web server | N6-methyladenosine-binding site | http://csbio.njust.edu.cn/bioinf/TargetM6A/ | Free |
| TS-m6A-DL [27] | 2021 | Web server | N6-methyladenosine-binding site | http://nsclbio.jbnu.ac.kr/tools/TS-m6A-DL/ | NA |
| VitaPred [28] | 2013 | Web server | Vitamin interacting residues | http://crdd.osdd.net/raghava/vitapred/ | Free |
| GTP-TP-RBF [29] | 2016 | Web server | GTP-binding site | http://140.138.155.226/~kahn/gtp-tp/ | NA |
| FAD-ETC-RBF [30] | 2016 | Web server | FAD(flavin adenine dinucleotide)-binding site | http://140.138.155.226/~kahn/Bioinformatics/ | NA |
| HEMEsPred [31] | 2018 | Web server | Heme-binding site | http://www.inforstation.com/HEMEsPred/ | Free |
| SAMbinder [32] | 2020 | Web server | S-adenosyl-L-methionine (SAM) | https://webs.iiitd.edu.in/raghava/sambinder/ | FOSS |

Table S2 General-purpose methods in the identification of binding sites

| Names | Year | Methods | Types | URL | Available |
|---|---|---|---|---|---|
| ConCavity [33] | 2009 | Sequence-based | Web server | http://compbio.cs.princeton.edu/concavity/ | FOSS |
| MPLs-Pred [34] | 2019 | Sequence-based | Web server | http://icdtools.nenu.edu.cn/ | Free |
| FunFOLD [35] | 2011 | Template-based | Web server | http://www.reading.ac.uk/bioinf/FunFOLD/ | FOSS |
| COFACTOR [36] | 2012 | Template-based | Web server | https://zhanglab.ccmb.med.umich.edu/COFACTOR/ | Free |
| WebPDBinder [37] | 2013 | Template-based | Web server | http://pdbinder.bio.uniroma2.it/PDBinder | FOSS |
| GalaxySite [38] | 2014 | Template-based | Web server | http://galaxy.seoklab.org/site/ | Free |
| LIBRA [39] | 2015 | Template-based | Web server | http://www.computationalbiology.it/software/LIBRAv1.zip/ | FOSS |
| LIBRA-WA [40] | 2017 | Template-based | Web server | http://biochimica3.bio.uniroma3.it/LIBRAWA/ | NA |
| Sitemap [41] | 2009 | Geometric-based | Software | https://www.schrodinger.com/products/sitemap/ | FOSS |
| MSPocket [42] | 2010 | Geometric-based | Web server | http://appserver.biotec.tu-dresden.de/MSPocket/ | FOSS |
| Fpocket [43] | 2010 | Geometric-based | Web server/software | http://www.sourceforge.net/projects/fpocket/ | FOSS |
| LISE [44] | 2013 | Geometric-based | Web server | http://lise.ibms.sinica.edu.tw/ | Free |
| Patch-Surfer2. 0 [45] | 2014 | Geometric-based | Web server | http://kiharalab.org/patchsurfer2.0/ | NA |
| CB-Dock [46] | 2019 | Geometric-based | Web server | http://cao.labshare.cn/cb-dock/ | Free |
| SITEHOUND [47] | 2009 | Energetic-based | Web server | http://sitehound.sanchezlab.org/ | NA |
| FTSite [48] | 2012 | Energetic-based | Web server | https://ftsite.bu.edu/ | Free |
| SiteComp [49] | 2012 | Energetic-based | Web server | http://sitecomp.sanchezlab.org/ | Free |
| MetaPocket 2.0 [50] | 2011 | Consensus-based | Web server | http://projects.biotec.tu-dresden.de/metapocket/ | Free |
| COACH [51] | 2013 | Consensus-based | Web server | http://zhanglab.ccmb.med.umich.edu/COACH/ | FOSS |
| DeepSite [52] | 2017 | Machine learning | Web server | https://playmolecule.com/deepsite/ | Free |
| COACH-D [53] | 2018 | Machine learning | Web server | http://yanglab.nankai.edu.cn/COACH-D/ | Free |
| P2Rank [54] | 2018 | Machine learning | Web server | http://siret.ms.mff.cuni.cz/p2rank/ | FOSS |
| BIPSPI [55] | 2018 | Machine learning | Web server | http://bipspi.cnb.csic.es/ | FOSS |
| Taba [56] | 2019 | Machine learning | Web server | https://github.com/azevedolab/taba/ | FOSS |
| DeepBind [57] | 2015 | Deep learning | Web server | http://tools.genes.toronto.edu/deepbind/ | FOSS |
| DeepDTA [58] | 2018 | Deep learning | Web server | https://github.com/hkmztrk/DeepDTA/ | FOSS |
| KDEEP [59] | 2018 | Deep learning | Web server | https://playmolecule.com/Kdeep/ | NA |
| DeepCSeqSite [60] | 2019 | Deep learning | Web server | https://github.com/yfCuiFaith/DeepCSeqSite/ | FOSS |
| DeepConv-DTI [61] | 2019 | Deep learning | Web server | https://github.com/GIST-CSBL/DeepConv-DTI/ | FOSS |
| DeepDrug3D [62] | 2019 | Deep learning | Web server | https://github.com/pulimeng/DeepDrug3D/ | FOSS |
| Onionnet [63] | 2019 | Deep learning | Web server | http://github.com/zhenglz/onionnet/ | FOSS |
| PUResNet [64] | 2021 | Deep learning | Web server | https://github.com/jivankandel/PUResNet/ | FOSS |

Table S3 Software or tools in the assessment of druggability

| Name | Methods | Types | Pocket search method | Druggabable criteria | URL | Avaible |
|---|---|---|---|---|---|---|
| PockDrug [65] | Structured-based | Web server | Prox and Fpocket | 0.87 ± 0.15 score | http://pockdrug.rpbs.univ-paris-diderot.fr/cgi-bin/index.py?page=home/ | FOSS |
| FTMap [66] | Hotspot-based | Web server | Solvent mapping algorithm | S>16 and the presence of at least one additional hotspot within 8 Å of a strong hotspot. | http://ftmap.bu.edu/login.php/ | Free |
| Sitemap [41] | Structured-based | Software | Geometric and energetic criteria on 3D grids | SiteScore>0.8 and Dscore>0.83 | https://www.schrodinger.com/products/sitemap/ | FOSS |
| Fpocket [67] | Structured-based | Software | Radii values between minimal and maximal in a-spheres. | 0.5–0.7(druggable); 0.7–1.0 (highly druggable) | http://www.sourceforge.net/projects/fpocket/ | FOSS |
| DoGSiteScorer [68] | Structured-based | Web server | 3D image enhancement techniques | A pocket volume of 1466 Å3, a depth of 19.27 Å as well as a polar amino acid ratio of 0.5 | https://www.biosolveit.de/DoGSiteScorer/ | FOSS |

**Reference**


1. Babor, M.; Gerzon, S.; Raveh, B.; Sobolev, V.; Edelman, M., Prediction of transition metal-binding sites from apo protein structures. *Proteins: Structure, Function, and Bioinformatics* **2008,** 70, (1), 208-217.
2. Brylinski, M.; Skolnick, J., FINDSITE-metal: Integrating evolutionary information and machine learning for structure-based metal-binding site prediction at the proteome level. *Proteins: Structure, Function, and Bioinformatics* **2011,** 79, (3), 735-751.
3. Hu, X.; Dong, Q.; Yang, J.; Zhang, Y., Recognizing metal and acid radical ion-binding sites by integrating ab initio modeling with template-based transferals. *Bioinformatics* **2016,** 32, (21), 3260-3269.
4. Chauhan, J. S.; Mishra, N. K.; Raghava, G. P. S., Identification of ATP binding residues of a protein from its primary sequence. *BMC Bioinformatics* **2009,** 10, (1), 434.
5. Chen, K.; Mizianty, M. J.; Kurgan, L., ATPsite: sequence-based prediction of ATP-binding residues. *Proteome Science* **2011,** 9, (1), S4.
6. Yu, D.-J.; Hu, J.; Huang, Y.; Shen, H.-B.; Qi, Y.; Tang, Z.-M.; Yang, J.-Y., TargetATPsite: A template-free method for ATP-binding sites prediction with residue evolution image sparse representation and classifier ensemble. *Journal of Computational Chemistry* **2013,** 34, (11), 974-985.
7. Hu, J.; Li, Y.; Zhang, Y.; Yu, D.-J., ATPbind: Accurate Protein–ATP Binding Site Prediction by Combining Sequence-Profiling and Structure-Based Comparisons. *Journal of Chemical Information and Modeling* **2018,** 58, (2), 501-510.
8. Si, J.; Zhang, Z.; Lin, B.; Schroeder, M.; Huang, B., MetaDBSite: a meta approach to improve protein DNA-binding sites prediction. *BMC Systems Biology* **2011,** 5, (1), S7.
9. Ma, X.; Guo, J.; Liu, H. D.; Xie, J. M.; Sun, X., Sequence-Based Prediction of DNA-Binding Residues in Proteins with Conservation and Correlation Information. *IEEE/ACM Transactions on Computational Biology and Bioinformatics* **2012,** 9, (6), 1766-1775.
10. Hu, J.; Li, Y.; Zhang, M.; Yang, X.; Shen, H. B.; Yu, D. J., Predicting Protein-DNA Binding Residues by Weightedly Combining Sequence-Based Features and Boosting Multiple SVMs. *IEEE/ACM Transactions on Computational Biology and Bioinformatics* **2017,** 14, (6), 1389-1398.
11. Yan, J.; Kurgan, L., DRNApred, fast sequence-based method that accurately predicts and discriminates DNA- and RNA-binding residues. *Nucleic Acids Research* **2017,** 45, (10), e84.
12. Chen, K.; Mizianty, M. J.; Kurgan, L., Prediction and analysis of nucleotide-binding residues using sequence and sequence-derived structural descriptors. *Bioinformatics* **2012,** 28, (3), 331-341.
13. Yu, D. J.; Hu, J.; Yang, J.; Shen, H. B.; Tang, J.; Yang, J. Y., Designing Template-Free Predictor for Targeting Protein-Ligand Binding Sites with Classifier Ensemble and Spatial Clustering. *IEEE/ACM Transactions on Computational Biology and Bioinformatics* **2013,** 10, (4), 994-1008.
14. Hu, J.; He, X.; Yu, D.-J.; Yang, X.-B.; Yang, J.-Y.; Shen, H.-B., A New Supervised Over-Sampling Algorithm with Application to Protein-Nucleotide Binding Residue Prediction. *PLoS One* **2014,** 9, (9), e107676.
15. Hu, J.; Li, Y.; Yan, W.-X.; Yang, J.-Y.; Shen, H.-B.; Yu, D.-J., KNN-based dynamic query-driven sample rescaling strategy for class imbalance learning. *Neurocomputing* **2016,** 191, 363-373.
16. Zhao, Z.; Xu, Y.; Zhao, Y., SXGBsite: Prediction of Protein–Ligand Binding Sites Using Sequence Information and Extreme Gradient Boosting. *Genes* **2019,** 10, (12), 965.
17. Xia, Y.; Xia, C.-Q.; Pan, X.; Shen, H.-B., GraphBind: protein structural context embedded rules learned by hierarchical graph neural networks for recognizing nucleic-acid-binding residues. *Nucleic Acids Research* **2021,** 49, (9), e51.
18. Khamis, A. M.; Motwalli, O.; Oliva, R.; Jankovic, B. R.; Medvedeva, Y. A.; Ashoor, H.; Essack, M.; Gao, X.; Bajic, V. B., A novel method for improved accuracy of transcription factor binding site prediction. *Nucleic Acids Research* **2018,** 46, (12), e72.
19. Zhou, J.; Lu, Q.; Gui, L.; Xu, R.; Long, Y.; Wang, H., MTTFsite: cross-cell type TF binding site prediction by using multi-task learning. *Bioinformatics* **2019,** 35, (24), 5067-5077.
20. Li, H.; Quang, D.; Guan, Y., Anchor: trans-cell type prediction of transcription factor binding sites. *Genome Research* **2019,** 29, (2), 281-292.
21. Quang, D.; Xie, X., FactorNet: A deep learning framework for predicting cell type specific transcription factor binding from nucleotide-resolution sequential data. *Methods* **2019,** 166, 40-47.
22. Li, H.; Guan, Y., Fast decoding cell type-specific transcription factor binding landscape at single-nucleotide resolution. *Genome Research* **2021,** 31, (4), 721-731.

23. Chen, C.; Hou, J.; Shi, X.; Yang, H.; Birchler, J. A.; Cheng, J., DeepGRN: prediction of transcription factor binding site across cell-types using attention-based deep neural networks. *BMC Bioinformatics* **2021,** 22, (1), 38.
24. Wang, H.; Zhao, Y., RBinds: A user-friendly server for RNA binding site prediction. *Computational and Structural Biotechnology Journal* **2020,** 18, 3762-3765.
25. Yang, Y.; Hou, Z.; Ma, Z.; Li, X.; Wong, K.-C., iCircRBP-DHN: identification of circRNA-RBP interaction sites using deep hierarchical network. *Briefings in Bioinformatics* **2021,** 22, (4), bbaa274.
26. Li, G. Q.; Liu, Z.; Shen, H. B.; Yu, D. J., TargetM6A: Identifying N6-Methyladenosine Sites From RNA Sequences via Position-Specific Nucleotide Propensities and a Support Vector Machine. *IEEE Transactions on NanoBioscience* **2016,** 15, (7), 674-682.
27. Abbas, Z.; Tayara, H.; Zou, Q.; Chong, K. T., TS-m6A-DL: Tissue-specific identification of N6-methyladenosine sites using a universal deep learning model. *Computational and Structural Biotechnology Journal* **2021,** 19, 4619-4625.
28. Panwar, B.; Gupta, S.; Raghava, G. P. S., Prediction of vitamin interacting residues in a vitamin binding protein using evolutionary information. *BMC Bioinformatics* **2013,** 14, (1), 44.
29. Le, N.-Q.-K.; Ou, Y.-Y., Incorporating efficient radial basis function networks and significant amino acid pairs for predicting GTP binding sites in transport proteins. *BMC Bioinformatics* **2016,** 17, (19), 501.
30. Le, N.-Q.-K.; Ou, Y.-Y., Prediction of FAD binding sites in electron transport proteins according to efficient radial basis function networks and significant amino acid pairs. *BMC Bioinformatics* **2016,** 17, (1), 298.
31. Zhang, J.; Chai, H.; Gao, B.; Yang, G.; Ma, Z., HEMEsPred: Structure-Based Ligand-Specific Heme Binding Residues Prediction by Using Fast-Adaptive Ensemble Learning Scheme. *IEEE/ACM Transactions on Computational Biology and Bioinformatics* **2018,** 15, (1), 147-156.
32. Agrawal, P.; Mishra, G.; Raghava, G. P. S., SAMbinder: A Web Server for Predicting S-Adenosyl-L-Methionine Binding Residues of a Protein From Its Amino Acid Sequence. *Frontiers in Pharmacology* **2020,** 10, 1690.
33. Capra, J. A.; Laskowski, R. A.; Thornton, J. M.; Singh, M.; Funkhouser, T. A., Predicting Protein Ligand Binding Sites by Combining Evolutionary Sequence Conservation and 3D Structure. *PLoS Computational Biology* **2009,** 5, (12), e1000585.
34. Lu, C.; Liu, Z.; Zhang, E.; He, F.; Ma, Z.; Wang, H., MPLs-Pred: Predicting Membrane Protein-Ligand Binding Sites Using Hybrid Sequence-Based Features and Ligand-Specific Models. *International Journal of Molecular Sciences* **2019,** 20, (13), 3120.
35. Roche, D. B.; Tetchner, S. J.; McGuffin, L. J., FunFOLD: an improved automated method for the prediction of ligand binding residues using 3D models of proteins. *BMC Bioinformatics* **2011,** 12, (1), 160.
36. Roy, A.; Yang, J.; Zhang, Y., COFACTOR: an accurate comparative algorithm for structure-based protein function annotation. *Nucleic Acids Research* **2012,** 40, (W1), W471-W477.
37. Bianchi, V.; Mangone, I.; Ferrè, F.; Helmer-Citterich, M.; Ausiello, G., webPDBinder: a server for the identification of ligand binding sites on protein structures. *Nucleic Acids Research* **2013,** 41, (W1), W308-W313.
38. Heo, L.; Shin, W.-H.; Lee, M. S.; Seok, C., GalaxySite: ligand-binding-site prediction by using molecular docking. *Nucleic Acids Research* **2014,** 42, (W1), W210-W214.
39. Viet Hung, L.; Caprari, S.; Bizai, M.; Toti, D.; Polticelli, F., LIBRA: LIgand Binding site Recognition Application. *Bioinformatics* **2015,** 31, (24), 4020-4022.
40. Toti, D.; Viet Hung, L.; Tortosa, V.; Brandi, V.; Polticelli, F., LIBRA-WA: a web application for ligand binding site detection and protein function recognition. *Bioinformatics* **2018,** 34, (5), 878-880.
41. Halgren, T. A., Identifying and Characterizing Binding Sites and Assessing Druggability. *Journal of Chemical Information and Modeling* **2009,** 49, (2), 377-389.
42. Zhu, H.; Pisabarro, M. T., MSPocket: an orientation-independent algorithm for the detection of ligand binding pockets. *Bioinformatics* **2011,** 27, (3), 351-358.
43. Schmidtke, P.; Le Guilloux, V.; Maupetit, J.; Tufféry, P., fpocket: online tools for protein ensemble pocket detection and tracking. *Nucleic Acids Research* **2010,** 38, (suppl_2), W582-W589.
44. Xie, Z.-R.; Liu, C.-K.; Hsiao, F.-C.; Yao, A.; Hwang, M.-J., LISE: a server using ligand-interacting and site-enriched protein triangles for prediction of ligand-binding sites. *Nucleic Acids Research* **2013,** 41, (W1), W292-W296.
45. Zhu, X.; Xiong, Y.; Kihara, D., Large-scale binding ligand prediction by improved patch-based method Patch-Surfer2.0. *Bioinformatics* **2015,** 31, (5), 707-713.
46. Liu, Y.; Grimm, M.; Dai, W.-t.; Hou, M.-c.; Xiao, Z.-X.; Cao, Y., CB-Dock: a web server for cavity detection-guided protein–ligand blind docking. *Acta Pharmacologica Sinica* **2020,** 41, (1), 138-144.

47. Hernandez, M.; Ghersi, D.; Sanchez, R., SITEHOUND-web: a server for ligand binding site identification in protein structures. *Nucleic Acids Research* **2009,** 37, (suppl_2), W413-W416.
48. Ngan, C.-H.; Hall, D. R.; Zerbe, B.; Grove, L. E.; Kozakov, D.; Vajda, S., FTSite: high accuracy detection of ligand binding sites on unbound protein structures. *Bioinformatics* **2012,** 28, (2), 286-287.
49. Lin, Y.; Yoo, S.; Sanchez, R., SiteComp: a server for ligand binding site analysis in protein structures. *Bioinformatics* **2012,** 28, (8), 1172-1173.
50. Zhang, Z.; Li, Y.; Lin, B.; Schroeder, M.; Huang, B., Identification of cavities on protein surface using multiple computational approaches for drug binding site prediction. *Bioinformatics* **2011,** 27, (15), 2083-2088.
51. Yang, J.; Roy, A.; Zhang, Y., Protein–ligand binding site recognition using complementary binding-specific substructure comparison and sequence profile alignment. *Bioinformatics* **2013,** 29, (20), 2588-2595.
52. Jiménez, J.; Doerr, S.; Martínez-Rosell, G.; Rose, A. S.; De Fabritiis, G., DeepSite: protein-binding site predictor using 3D-convolutional neural networks. *Bioinformatics* **2017,** 33, (19), 3036-3042.
53. Wu, Q.; Peng, Z.; Zhang, Y.; Yang, J., COACH-D: improved protein–ligand binding sites prediction with refined ligand-binding poses through molecular docking. *Nucleic Acids Research* **2018,** 46, (W1), W438-W442.
54. Krivák, R.; Hoksza, D., P2Rank: machine learning based tool for rapid and accurate prediction of ligand binding sites from protein structure. *Journal of Cheminformatics* **2018,** 10, (1), 39.
55. Sanchez-Garcia, R.; Sorzano, C. O. S.; Carazo, J. M.; Segura, J., BIPSPI: a method for the prediction of partner-specific protein–protein interfaces. *Bioinformatics* **2019,** 35, (3), 470-477.
56. da Silva, A. D.; Bitencourt-Ferreira, G.; de Azevedo Jr, W. F., Taba: A Tool to Analyze the Binding Affinity. *Journal of Computational Chemistry* **2020,** 41, (1), 69-73.
57. Alipanahi, B.; Delong, A.; Weirauch, M. T.; Frey, B. J., Predicting the sequence specificities of DNA- and RNA-binding proteins by deep learning. *Nature Biotechnology* **2015,** 33, (8), 831-838.
58. Öztürk, H.; Özgür, A.; Ozkirimli, E., DeepDTA: deep drug–target binding affinity prediction. *Bioinformatics* **2018,** 34, (17), i821-i829.
59. Jiménez, J.; Škalič, M.; Martínez-Rosell, G.; De Fabritiis, G., KDEEP: Protein–Ligand Absolute Binding Affinity Prediction via 3D-Convolutional Neural Networks. *Journal of Chemical Information and Modeling* **2018,** 58, (2), 287-296.
60. Cui, Y.; Dong, Q.; Hong, D.; Wang, X., Predicting protein-ligand binding residues with deep convolutional neural networks. *BMC Bioinformatics* **2019,** 20, (1), 93.
61. Lee, I.; Keum, J.; Nam, H., DeepConv-DTI: Prediction of drug-target interactions via deep learning with convolution on protein sequences. *PLoS Computational Biology* **2019,** 15, (6), e1007129.
62. Pu, L.; Govindaraj, R. G.; Lemoine, J. M.; Wu, H.-C.; Brylinski, M., DeepDrug3D: Classification of ligand-binding pockets in proteins with a convolutional neural network. *PLoS Computational Biology* **2019,** 15, (2), e1006718.
63. Zheng, L.; Fan, J.; Mu, Y., OnionNet: a Multiple-Layer Intermolecular-Contact-Based Convolutional Neural Network for Protein–Ligand Binding Affinity Prediction. *ACS Omega* **2019,** 4, (14), 15956-15965.
64. Kandel, J.; Tayara, H.; Chong, K. T., PUResNet: prediction of protein-ligand binding sites using deep residual neural network. *Journal of Cheminformatics* **2021,** 13, (1), 65.
65. Hussein, H. A.; Borrel, A.; Geneix, C.; Petitjean, M.; Regad, L.; Camproux, A.-C., PockDrug-Server: a new web server for predicting pocket druggability on holo and apo proteins. *Nucleic Acids Research* **2015,** 43, (W1), W436-W442.
66. Ngan, C. H.; Bohnuud, T.; Mottarella, S. E.; Beglov, D.; Villar, E. A.; Hall, D. R.; Kozakov, D.; Vajda, S., FTMAP: extended protein mapping with user-selected probe molecules. *Nucleic Acids Research* **2012,** 40, (W1), W271-W275.
67. Le Guilloux, V.; Schmidtke, P.; Tuffery, P., Fpocket: An open source platform for ligand pocket detection. *BMC Bioinformatics* **2009,** 10, (1), 168.
68. Volkamer, A.; Kuhn, D.; Rippmann, F.; Rarey, M., DoGSiteScorer: a web server for automatic binding site prediction, analysis and druggability assessment. *Bioinformatics* **2012,** 28, (15), 2074-2075.
